# Holliday Cross-Recognition Protein *HJURP*: Association With the Tumor Microenvironment in Hepatocellular Carcinoma and With Patient Prognosis

**DOI:** 10.3389/pore.2022.1610506

**Published:** 2022-06-17

**Authors:** Dongcheng Luo, Sina Liao, Yu Liu, Youzhi Lin, Yongqiang Li, XiaoLi Liao

**Affiliations:** ^1^ Department of First Chemotherapy, Guangxi Medical University Cancer Hospital, Nanning, China; ^2^ Hepatobiliary Surgery Department, Guangxi Medical University Cancer Hospital, Nanning, China

**Keywords:** *HJURP*, hepatocellular carcinoma, tumor microenvironment, prognosis, GSEA, WGCNA, RNA-seq, single-cell RNA-seq

## Abstract

**Background:** Hepatocellular carcinoma is the most common type of primary liver cancer, and it is associated with poor prognosis. It often fails to respond to immunotherapy, highlighting the need to identify genes that are associated with the tumor microenvironment and may be good therapeutic targets. We and others have shown that the Holliday cross-recognition protein *HJURP* can promote the proliferation, migration, and invasion by hepatocellular carcinoma cells, and that *HJURP* overexpression is associated with poor survival. Here we explored the potential relationship between *HJURP* and the tumor microenvironment in hepatocellular carcinoma.

**Methods:** We used the Immuno-Oncology-Biological-Research (IOBR) software package to analyze the potential roles of *HJURP* in the tumor microenvironment. Using single-cell RNA sequencing data, we identified the cell clusters expressing abundant *HJURP*, then linked some of these clusters to certain bioprocesses using Gene Set Enrichment Analysis (GSEA). We validated the differential expression of *HJURP* in tumor-infiltrating CD8^+^ T cells, sorted by flow cytometry into populations based on the expression level of PD-1. We used weighted gene co-expression network analysis (WGCNA) to identify immunity-related genes whose expression strongly correlated with that of *HJURP*. The function of these genes was validated based on enrichment in Gene Ontology (GO) terms, and they were used to establish a prognosis prediction model.

**Results:** IOBR analysis suggested that *HJURP* is significantly related to the immunosuppressive tumor microenvironment and was significantly related to T cells, dendritic cells, and B cells. Based on single-cell RNA sequencing, *HJURP* was strongly expressed in T cells, erythrocytes, and B cells from normal liver tissues, as well as in CD8^+^ T cells, dendritic cells, and one cluster of hepatocytes in hepatocellular carcinoma tissues. Malignant hepatocytes strongly expressing *HJURP* were associated with the downregulation of immune bioprocesses. *HJURP* expression was significantly higher in CD8^+^ T cells strongly expressing PD-1 than in those expressing no or intermediate levels of PD1. WGCNA identified two module eigengenes (comprising 397 and 84 genes) related to the tumor microenvironment. We identified 24 hub genes and confirmed that they were related to immune regulation. A prognostic risk score model based on expression of *HJURP*, *PPT1*, *PML*, and *CLEC7A* showed moderate ability to predict survival.

**Conclusion:** *HJURP* is associated with tumor-infiltrating immune cells, immune checkpoints, and immune suppression in hepatocellular carcinoma. *HJURP*-related genes involved in immune responses may be useful for predicting patient prognosis.

## Introduction

Globally, primary liver cancer was the sixth most common cancer and the third leading cause of cancer deaths in 2020, giving rise to approximately 906,000 new cases and 830,000 deaths (1). Hepatocellular carcinoma (HCC) accounts for 75%–85% of primary liver cancer (1). If diagnosed early enough, HCC can be treated using surgical resection, liver transplantation, adjuvant radio- and chemotherapy, radiofrequency ablation, and interventional therapy (2). If diagnosed at an advanced stage, however, treatment options are limited. The median survival for patients with advanced HCC is only 6–20 months after diagnosis, and the average 5-year survival rate is less than 15% (3, 4).

Systemic treatment appears to be essential for patients with advanced HCC, and such treatments usually involve tyrosine kinase inhibitors, immune checkpoint inhibitors (ICIs), monoclonal antibodies against vascular endothelial growth factor, or chemotherapy. The American Society of Clinical Oncology recommends atezolizumab, bevacizumab, sorafenib, lenvatinib, and regorafenib as first- and second-line therapy, while the ICIs pembrolizumab and nivolumab are recommended as second-line options. However, only 14%–20% of patients with advanced HCC respond to pembrolizumab or nivolumab (5).

One of the obstacles to effective ICIs therapy may be the tumor microenvironment (TME) (6), which in HCC may induce immune tolerance and escape (7). Indeed, the characteristics of the TME may help explain why ICIs are less effective against HCC than against melanoma or non-small cell lung cancer. Therefore, exploring the genes related to the TME in HCC may not only clarify disease pathogenesis but also guide the development of more effective immunotherapies.

The Holliday cross-recognition protein *HJURP* is a molecular chaperone of the histone H3 variant Cenp-A. *HJURP* maintains Cenp-A on the centromere and participates in chromatin separation (8, 9). *HJURP* is also involved in DNA replication (10). As a result, *HJURP* can participate in various cell proliferation-related pathways and promote the proliferation of tumor cells (11–16). We previously reported that *HJURP* is more strongly expressed in HCC tissues than in adjacent normal tissues, and its high expression is a risk factor for poor prognosis in HCC patients (17). We also demonstrated that *HJURP* promotes HCC cell proliferation, migration, and invasion, while promoting progression through the cell cycle and apoptosis.

Given the tight association between *HJURP* and HCC pathogenesis and progression, we explored here whether the *HJURP* gene might be associated with the TME. Our results may help identify new ways to overcome the barriers to effective immunotherapy against advanced HCC.

## Materials and Methods

### Data Preparation and Preprocessing

We used UCSCXenaTools (18) to download bulkRNA sequence counts (transcriptomics analysis of pooled cell populations, tissue sections or biopsies) from the TCGA-LIHC cohort, extracting tumor data for 368 patients (Supplementary Table S1). In addition, RNA sequence counts for 232 patients in the LIRI-JP cohort were downloaded from the International Cancer Genome Consortium (ICGC) database (Supplementary Table S2). Finally, data were extracted from the GSE111389 and GSE156625 datasets in the GEO database (https://www.ncbi.nlm.nih.gov/gds/?term=GSE111389[Accession] & https://www.ncbi.nlm.nih.gov/gds/?term=GSE156625[Accession]). GSE111389 contains high-throughput sequencing of tumor-infiltrating lymphocytes (TILs) in HCC, including CD8^+^ T cells expressing no, intermediate or high levels of PD-1 (19). All high-throughput sequencing counts were normalized using the TPM method. Samples with low-frequency counts were excluded. Genes were excluded if their expression levels were below the lower end of the interquartile range (IQR) of expression across all genes in the sample.

### Differential Expression and Prognostic Value of *HJURP*

*HJURP* expression was compared between HCC and normal tissues using GEPIA2 (http://gepia2.cancer-pku.cn/). The survival analysis module in this software was used to perform univariable survival analysis. Then a multivariable Cox regression model was built using the “survival” package in R 4.1.0 (20) in order to examine whether *HJURP* expression was an independent prognostic factor.

### TME Analysis

The Immuno-Oncology-Biological-Research (IOBR) package in R integrates 6 commonly used algorithms (MCPcounter, TIMER, xCell, CIBERSORT, EPIC and quanTiseq) to separately analyze tumor-infiltrating immune cells (TILs) in the TME, and it draws on 255 gene signatures related to tumors and the TME (21). We calculated scores for TILs and for the TME signature in the TCGA-LIHC cohort, then we compared the scores between 92 samples whose *HJURP* expression was below the lower end of the IQR and 92 samples whose *HJURP* expression exceeded the upper end of the IQR.

### Single-Cell Sequencing Validation of *HJURP* Expression in Immune Cells and Other TME-Related Cells

Using data from the Human Protein Atlas (https://www.proteinatlas.org/), we compared *HJURP* expression between normal liver tissue and peripheral blood mononuclear cells (PBMCs) at the single-cell level. We also downloaded single-cell transcriptome sequencing data for 57 HCC specimens from the GSE156625 dataset (22), which we subjected to a standard preprocess workflow in Seurat 4.0.4 (23). We filtered out genes that were expressed in fewer than three cells, and we removed cells whose mitochondrial gene expression accounted for >5% of total gene expression as well as cells with “nFeature_RNA” below 200 and more than 5000. With the remaining 78,430 cells, we reduced the dimensionality of the data using principal component analysis (PCA). Based on Elbow analysis, we selected the first 15 principal components in order to cluster cells (Supplementary Figure S1). Marker genes for each cluster were identified using the Wilcoxon test (adjusted *p* < 0.05), then each cluster was annotated using the marker list (22). We visualized the clustering results using t-distributed stochastic neighbor embedding (TSNE). We used the Wilcoxon test to identify cell clusters strongly expressing *HJURP*. Gene Set Enrichment Analysis (GSEA) was performed on the cluster of *HJURP*-expressing malignant hepatocytes.

### Correlation Between *HJURP* Expression and Immune Checkpoints in HCC Based on RNA Sequencing

*HJURP* expression was compared across CD8^+^ T cells expressing negative, intermediate or high levels of PD-1. The TILs came from six HCC specimens in the GSE111389 dataset and had been sorted using fluorescence-activating cell sorting (FACS).

### Weighted Gene Co-Expression Network Analysis

Among the TCGA samples, we extracted the subset showing *HJURP* expression at levels higher than 75% of all samples, we excluded low-quality samples and outliers (Supplementary Figure S2A), then we performed weighted gene co-expression network analysis (WGCNA) using the WGCNA package in R (24). A scale-free network with power = 7 was constructed based on SFT.R.sq and mean connectivity (Supplementary Figure S2B). Module eigengenes (MEs) were identified, and those significantly related to *HJURP* expression (*p* < 0.05) were analyzed for potential correlation with the TME signature score. For the resulting TME-related module eigengenes, we calculated their gene significance (GS), defined as the absolute value of the correlation between the gene and the trait; eigengene connectivity (KME), defined as the degree of connection between a gene and other genes; and module membership (MM), defined as the correlation between module eigengenes and the gene expression profile. Finally, we screened for hub genes based on criteria of GS > 0.4 and KME >0.8, and we used Cytoscape (25) to visualize co-expression networks with vital edges in each module.

### Analysis of Enrichment in Gene Ontology Terms

We analyzed hub genes for their enrichment in Gene Ontology (GO) biological processes using the clusterprofiler package (26). We defined enrichment as an adjusted *p* < 0.05, where adjustment was performed using the Benjamini-Hochberg method. Results were visualized using enrichplot (27).

### Influence of *HJURP*- and Immune-Related Genes on Prognosis in HCC

We assessed the prognostic potential of hub genes using univariable Cox regression. Then we established the following risk score model using multivariable Cox regression. Variables were selected for Cox regression using forward selection based on the likelihood ratio (forward LR). The risk score was developed using the TCGA-LIHC cohort, and externally validated using the ICGC-LIRI-JP cohort. The final risk score was

risk score=(HJURP×0.09523)+(PPT1×0.04176)+(PML×−0.04464)+(CLEC7A×−0.1061)

The prognostic potential of the risk score and other prognostic parameters was assessed based on the area under time-dependent receiver operating characteristic curves (AUC). The four genes in the risk score were used to build a random survival forest model, whose tree number was set to 1000.

In the end, we developed a prognostic model based on the risk score, age, sex, grade, prothrombin time, and tumor stage. The ability of the model to predict survival was assessed using calibration curves and AUCs.

### Statistical Analysis

Data were analyzed statistically using R 4.1.0 (http://www.r-project.org) and SPSS 21 (IBM, Armonk, NY, United States). Differences between groups were assessed for significance using the Student-Newman-Keuls test, or the Wilcoxon test as appropriate. Potential correlations were assessed using Pearson coefficients. Overall survival was compared between groups using the Kaplan-Meier method and log-rank test.

Prognostic factors were assessed in terms of hazard ratios (HRs) and their 95% confidence intervals (CIs) based on univariable and multivariable Cox regression within the “survival” package in R. Time-dependent receiver operating characteristic curves and their AUCs were calculated using the “timeROC” package in R. Statistical significance was set at *p* < 0.05, and all *p* values were two-tailed.

## Results

### *HJURP* Expression Is Associated With HCC Pathogenesis and Patient Prognosis

**FIGURE 1:**
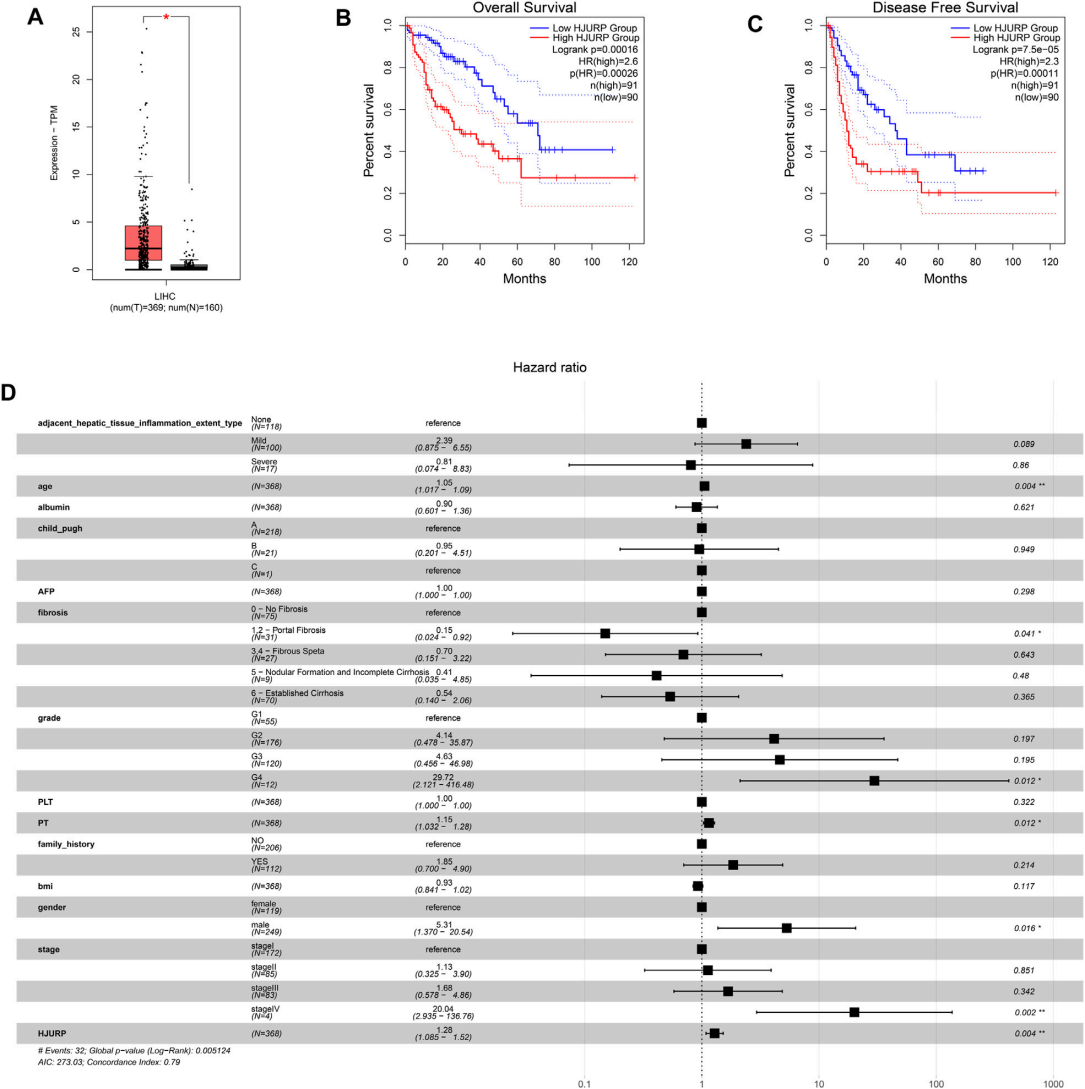
Expression analysis based on the GEPIA2 database, and prognosis analysis based on the TCGA LIHC cohort. **(A)** Comparison of *HJURP* expression between HCC tissue and normal liver. Kaplan-Meier survival analysis of **(B)** overall survival and **(C)** progression-free survival. Survival curves were compared using the log-rank test. **(D)** The forest plot shows a Cox regression model containing *HJURP* and other potential prognostic variables. *p < 0.05; **p < 0.01; ***p < 0.001; ns: no significance.

The GEPIA2 database showed that *HJURP* expression was significantly higher in HCC than normal tissues (*p* < 0.05; Figure 1A), and higher expression correlated with lower overall and progression-free survival (*p* < 0.001; Figures 1B,C). These results suggest that increased expression of *HJURP* may be a risk factor for poor prognosis.

A multivariable Cox regression model was defined using data on HCC patients from the TCGA-LIHC cohort, *HJURP* expression and other independent prognostic factors, including age, gender, Child-Pugh liver function classification, and tumor stage (Figure 1D). The HR for *HJURP* was 1.23 (*p* < 0.01), which confirmed that *HJURP* is an independent prognostic factor for HCC.

### *HJURP* Is Associated With TME Signatures and Immune Cell Infiltration Signatures

**FIGURE 2:**
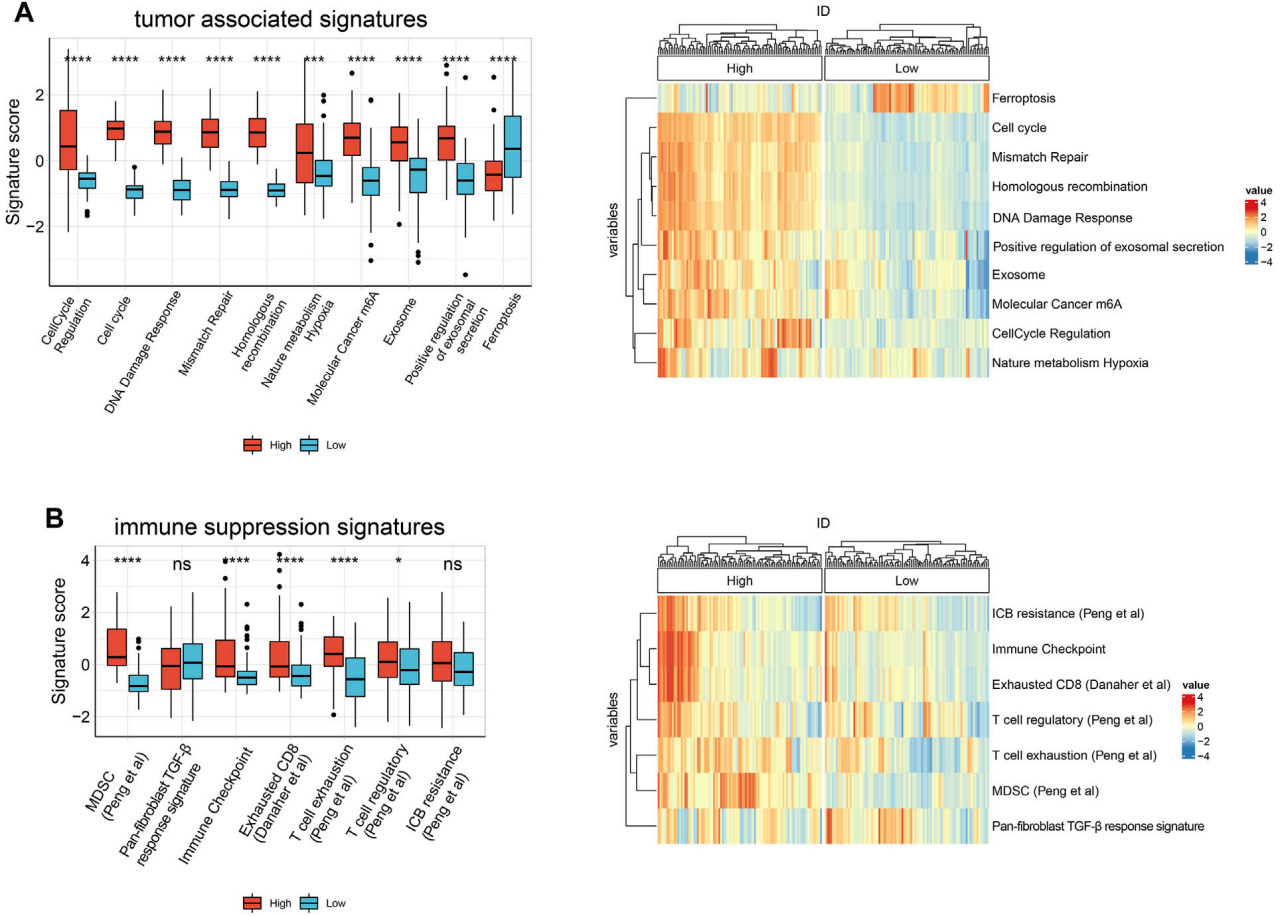
Boxplot of the results of IOBR analysis comparing samples showing high or low *HJURP* expression in terms of **(A)** tumor associated signatures **(B)** immune suppression signatures. *p < 0.05, **p < 0.01, *** <0.001, ****p < 0.0001.

To further explore whether *HJURP* is associated with the TME in HCC, we used IOBR to define TME signatures. We found that tumor-related signature scores were significantly higher in samples expressing high *HJURP* than in samples expressing low *HJURP*. These signatures included genes involved in cell cycle regulation, cell cycle, DNA damage repair (DDR), mismatch repair, and homogeneous recombination, nature metabolism hypoxia, molecular cancer m6A, exosome, positive regulation of exosomal secretion (Figure 2A). Contrary to the above signatures, ferroptosis scores was significantly lower in samples expressing high *HJURP* (Figure 2A). Scores for TME-related signatures for myeloid-derived suppressor cells (MDSCs), immune checkpoints, and CD8^+^ T cell depletion were also significantly higher in samples overexpressing *HJURP* than in those not overexpressing it (Figure 2B). These results link high *HJURP* expression with high scores for TME-related signatures, which indicates a more immunosuppressive TME.

**FIGURE 3:**
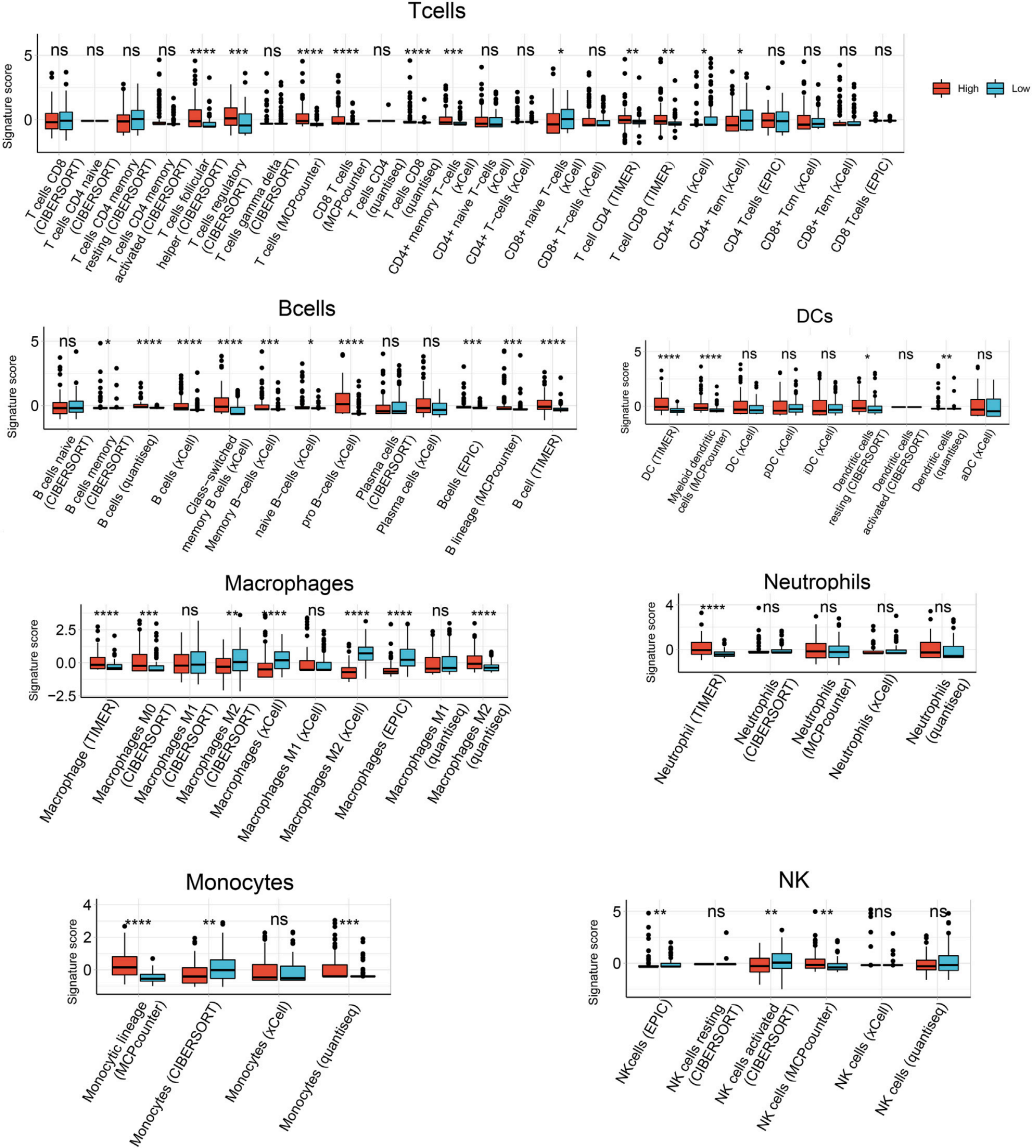
Analysis of tumor-infiltrating immune cells based on six prediction algorithms: TIMER, quanTIseq, MCPcounter, xCell, CIBERSORT, EPIC. *p < 0.05, **p < 0.01, ***p < 0.001, ****p < 0.0001.

Next we used six algorithms (MCPcounter, quanTiseq, xCell, CIBERSORT, EPIC and TIMER) to estimate immune cell infiltration in HCC. Infiltration by B cells was significantly higher in samples expressing high *HJURP* than in samples expressing low *HJURP,* except for naive B cells as calculated by CIBERSORT and plasma cells as calculated by CIBERSORT and xCell (Figure 3).

Infiltration by follicular helper T cells and regulatory T cells, as calculated by CIBERSORT, was significantly higher in samples expressing high *HJURP* than in samples expressing low *HJURP*. Infiltration by CD8^+^ T cells, as calculated by MCPcounter, quanTIseq, and TIMER, were significantly higher in samples expressing high *HJURP.* Some T cell subtypes showed a tendency toward higher infiltration in samples expressing high *HJURP,* as calculated by xCell, but this tendency was not as strong as the difference calculated by other algorithms (Figure 3).

Infiltration by dendritic cells (DCs), as calculated by TIMER, MCPcounter, CIBERSORT, and quanTIseq, was significantly higher in samples expressing high *HJURP*. Different algorithms gave different results for infiltration by macrophages. Infiltration was significantly higher in samples expressing high *HJURP* in the case of macrophages, as calculated by TIMER; M0 macrophages, as calculated by CIBERSORT; M2 macrophages, as calculated by quanTIseq; and M2 macrophages, as calculated by CIBERSORT. Conversely, infiltration was significnatly lower in samples expressing high *HJURP* in the case of macrophages, as calculated by xCell and EPIC; and M2 macrophages, as calculated by xCell (Figure 3).

Infiltration by neutrophils did not differ significantly between samples showing low or high *HJURP* expression, except when analyzed using TIMER. The various algorithms gave conflicting results for monocytes and natural killer cells (Figure 3).

In general, the various algorithms gave consistent results for all types of B cells, some types of T cells, and dendritic cells: their infiltration was higher in samples expressing high *HJURP* (Figure 3).

### *HJURP* is Highly Expressed in Immune Cells and Malignant Liver Cells

**FIGURE 4:**
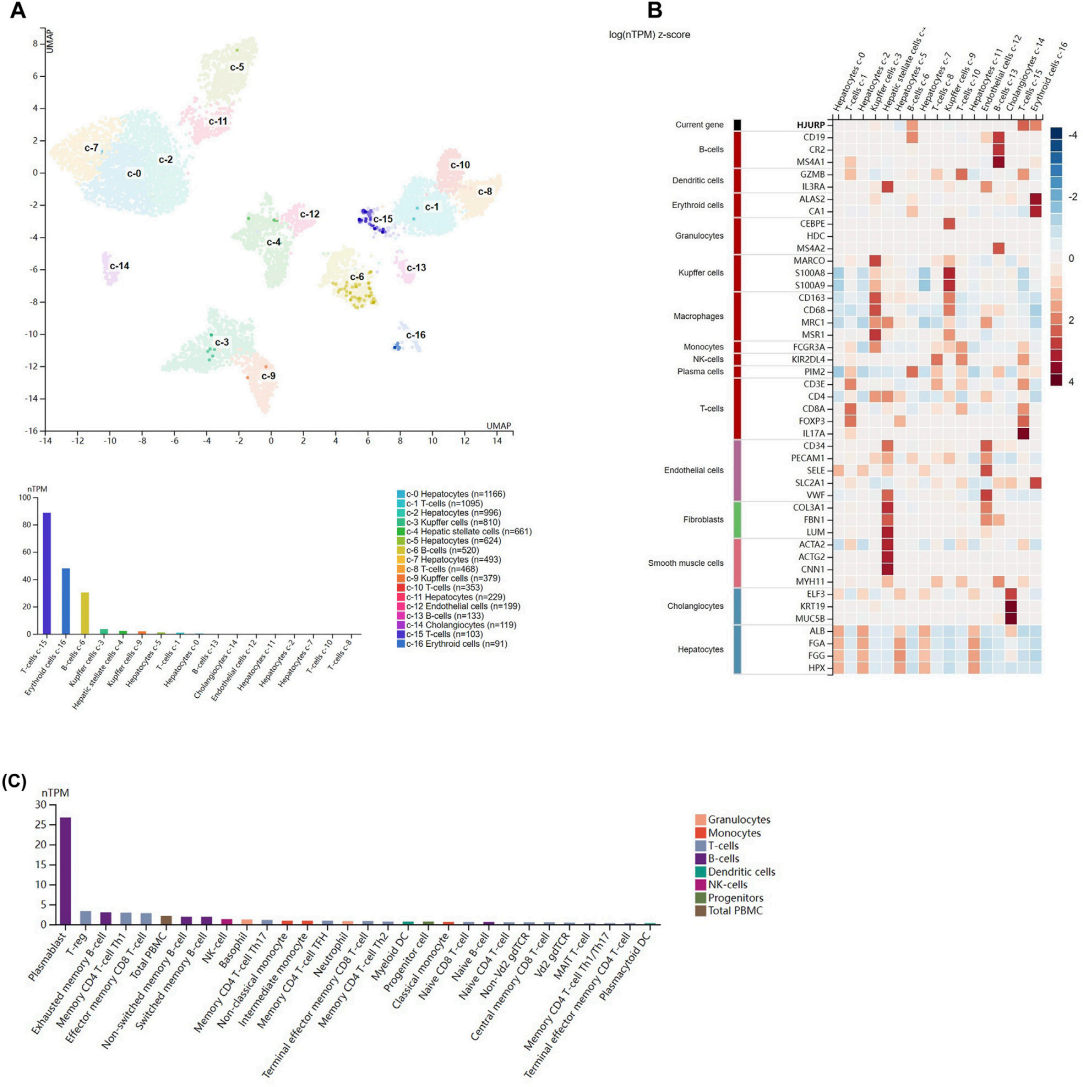
Analysis of *HJURP* expression at the single-cell level. **(A)** Clusters were defined based on *HJURP* expression in normal liver, then visualized using a UMAP plot and a bar chart. **(B)** Heatmap of the expression of *HJURP* and well-known cell type markers in the different clusters of normal liver. The panel on the left defines the markers associated with each cluster. Cell types are color-coded. **(C)** TPM-normalized *HJURP* expression in 29 types of blood cell and total peripheral blood mononuclear cells (PBMCs).

Single-cell data from the Human Protein Atlas showed that *HJURP* was highly expressed in T cells (c-15), erythrocytes(c-16), and B cells (c-6) in normal liver (Figure 4A). Figure 4B shows the expression of marker genes of various cell clusters in the normal liver. In PBMCs, *HJURP* was highly expressed in plasmablasts, Treg cells, exhausted memory B cells, memory CD4 Th1 T cells, CD8^+^ memory effector T cells and other cells (Figure 4C).

**FIGURE 5:**
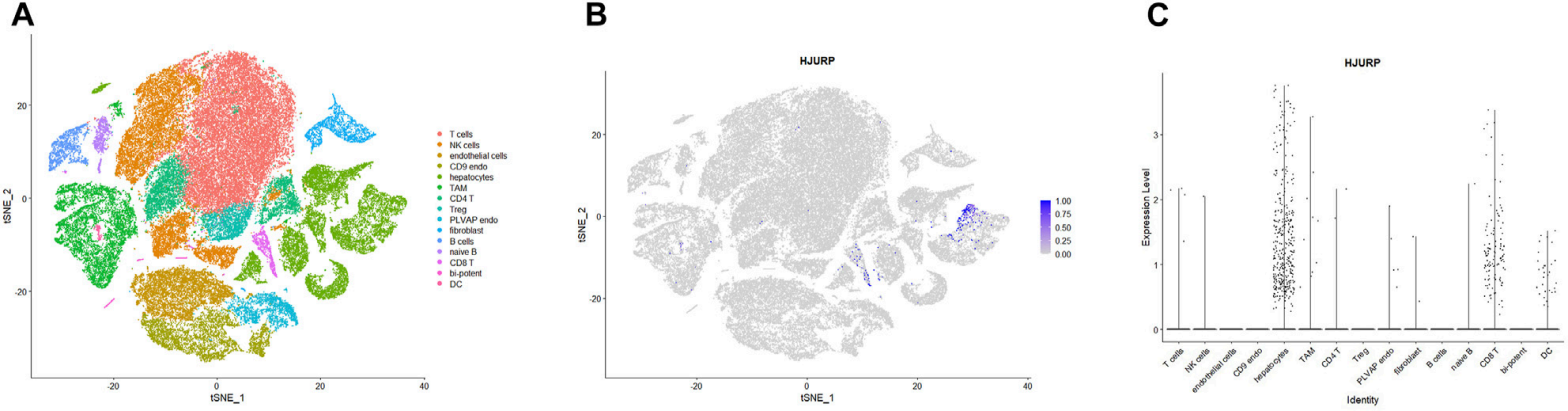
**(A)** Clustering and annotation of single-cell RNA sequencing data in the GSE156625 database, visualized by TSNE plot. **(B, C)** *HJURP* expression in clusters in HCC, visualized by TSNE and scatter plots.

To further explore which cell clusters highly express *HJURP* in the HCC TME, we processed single-cell RNA sequencing data from HCC tissues using the standard Seurat procedure and obtained 15 cell clusters (Figure 5A). The expression of marker genes in each cell cluster are shown in Supplementary Table S3. *HJURP* was highly expressed in CD8^+^ T cells, dendritic cells, and hepatocytes (Figures 5B,C; Supplementary Table S4), which verified the correlation between *HJURP* and tumor-infiltrating immune cells. *HJURP* was highly expressed in the hepatocytes of HCC tissues, most of which are malignant, but not in hepatocytes of normal liver. These results suggest that the observed overexpression of *HJURP* in tumor tissues may reflect overexpression in malignant hepatocytes.

### Downregulation of Immune-Related Genes in Hepatocytes Strongly Expressing *HJURP*

**FIGURE 6:**
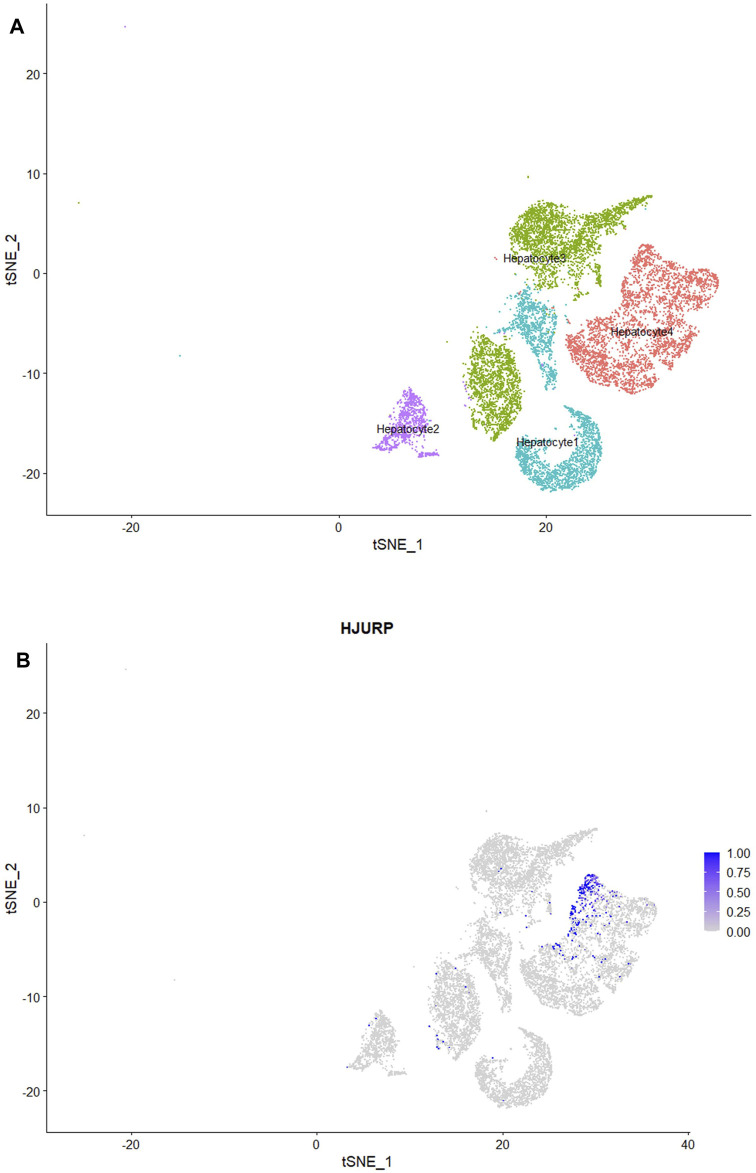
**(A)** HCC hepatocytes after classification into four subclusters, visualized by TSNE plot. **(B)** Subclusters strongly expressing *HJURP*, visualized by TSNE plot.

**FIGURE 7:**
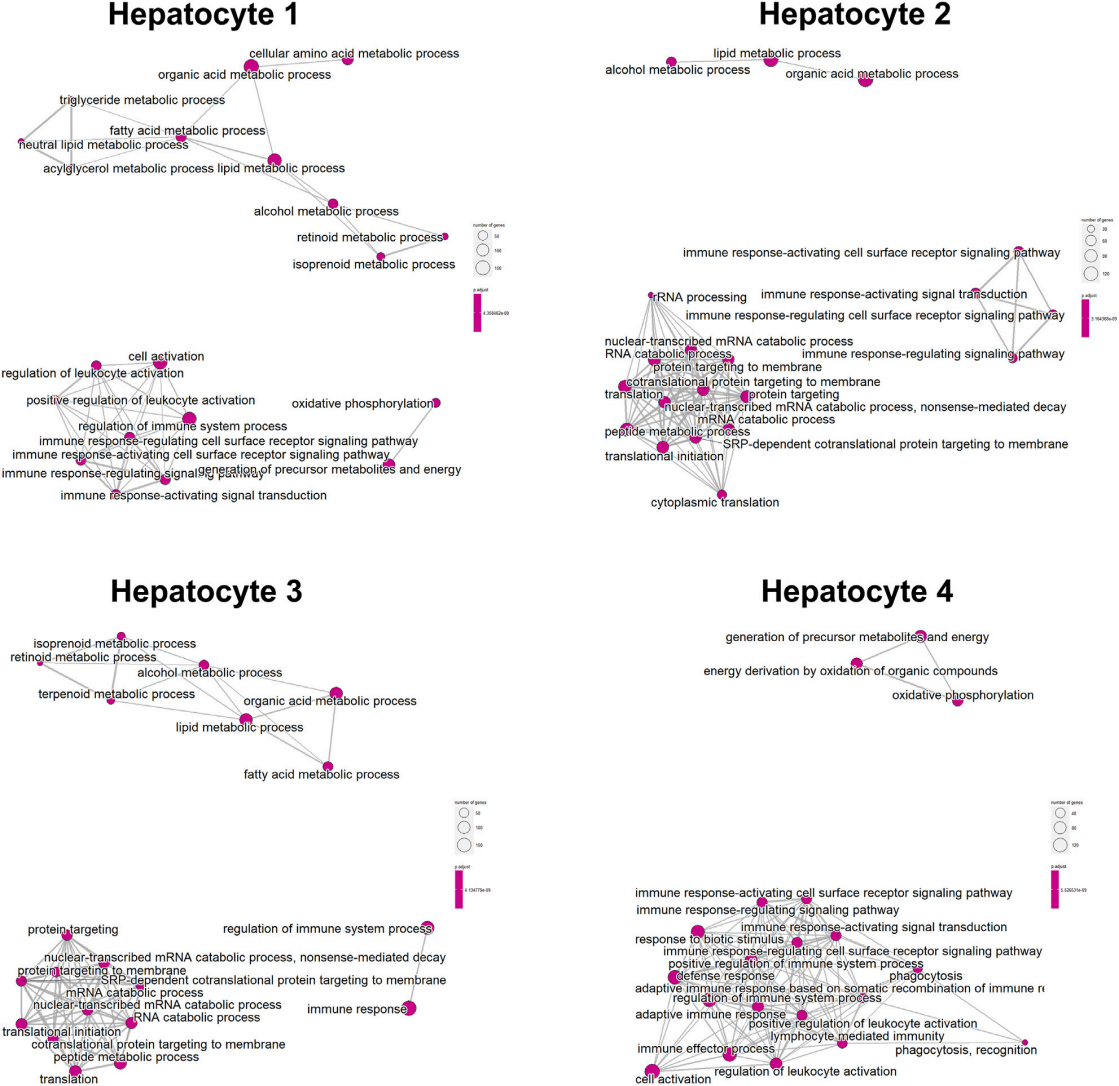
Enrichment map of the clusters Hepatocytes1, Hepatocytes2, Hepatocytes3, and Hepatocytes4 based on GSEA.

**FIGURE 8:**
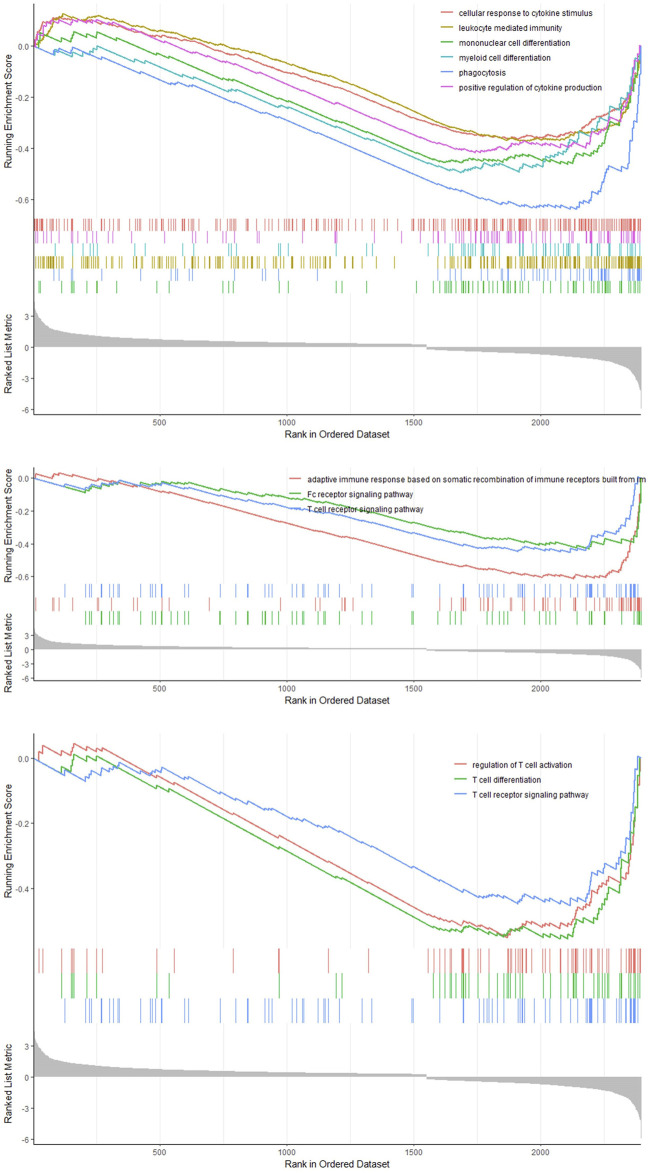
GSEA curve in the cluster Hepatocytes4.

We classified the hepatocytes into four clusters and annotated them using a marker list (Figure 6A). *HJURP* expression was higher in the cluster Hepatocytes4 than in other clusters (Figure 6B). We used GSEA to analyze biological processes occurring in each hepatocyte cluster (Figure 7). The cluster Hepatocytes1 was found to be related mainly in metabolism and immune-related biological processes; Hepatocytes2 and Hepatocytes3, metabolism and processes related to the genetic central dogma; and Hepatocytes4, immune processes and processes related to oxidative respiratory chain regulation. Most biological processes in Hepatocytes4 were related to immune responses, in contrast to other clusters, so we focused on Hepatocytes4 as an important immune-related cluster. GSEA enrichment scores were negative for non-specific and specific immune response biological processes in this cluster, suggesting downregulation of immune-related biological processes (Figure 8).

### *HJURP* May Be Related to CD8^+^ T Cell Immune Checkpoints

**FIGURE 9:**
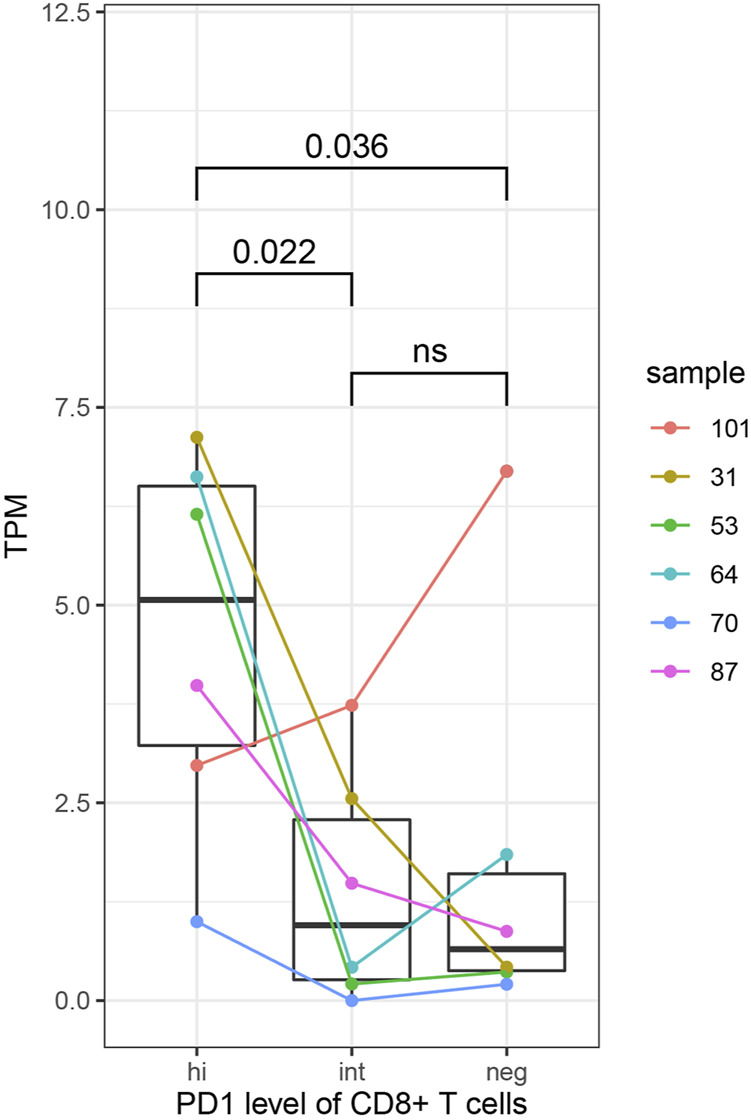
*HJURP* expression analysis in subsets of CD8^+^ T cells expressing negative, intermediate or high levels of PD-1. Differences were assessed for significance using the Student-Newman-Keuls test.

In order to further explore the relationship between immune molecular characteristics of CD8^+^ T cells and expression of *HJURP*, we compared the expression of *HJURP* in CD8^+^ T cells expressing negative, intermediate or high PD-1. *HJURP* expression was significantly higher in CD8^+^ T cells expressing high levels of PD-1 (*p* < 0.05; Figure 9). These results implicate *HJURP* in immune checkpoints.

### Association of TME-Related Module Eigengenes With *HJURP*

A gene co-expression network based on data from samples strongly expressing *HJURP* was built up and classified into 95 module eigengenes (MEs) (Supplementary Figure S2C). *HJURP*-related module eigengenes were defined as those correlating with *HJURP* expression, based on a correlation *p* < 0.05. Among *HJURP*-related module eigengenes, MEcyan and MEgreenyellow were found to be associated with TME signatures (Supplementary Figure S3), and their expression correlated positively with hepatic tissue inflammation and liver fibrosis (*p* < 0.01; Supplementary Figure S4). There was also a tendency for MEcyan to be associated with survival time, though the correlation did not achieve significance. Scatter plots showed good correlations of GS with MM and KME (Supplementary Figure S5).

Relationships among MEcyan, MEgreenyellow, and TME signatures are shown in Supplementary Figure S6. A total of 27 hub genes were screened out from MEcyan, but none from MEgreenyellow. Scatter plots and Pearson correlation analyses confirmed correlations between *HJURP* and all hub genes except *TCHH* (Supplementary Figure S7). Therefore we excluded *TCHH* as well as two genes encoding long non-coding RNAs (*LINC01094*, *L3MBTL4−AS1*), leaving 24 hub genes for subsequent analysis. Cytoscape was used to depict parts of the network based on MEcyan and MEgreenyellow (Supplementary Figure S8).

### Hub Genes May Be Involved in Immune Regulation

**FIGURE 10:**
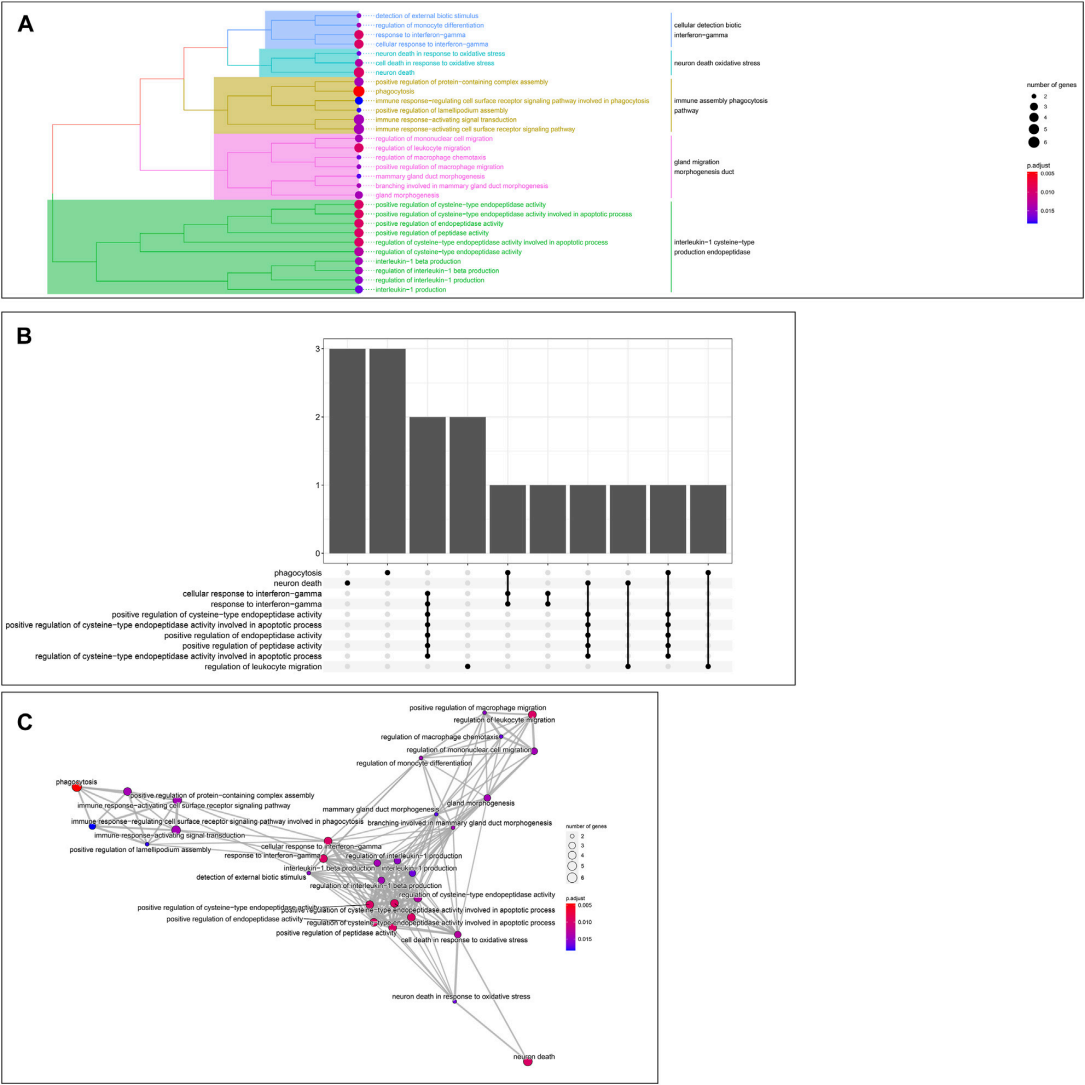
Enrichment in Gene Ontology (GO) biological processes. **(A)** Tree plot showing hierarchical clustering of enriched terms, based on Jaccard’s similarity index. Terms were aggregated by ward. D method **(B)** Upsetplot showing complex associations between genes and gene sets. **(C)** Enrichment map showing the arrangement of enriched terms in a network whose edges connect overlapping gene sets.

**FIGURE 11:**
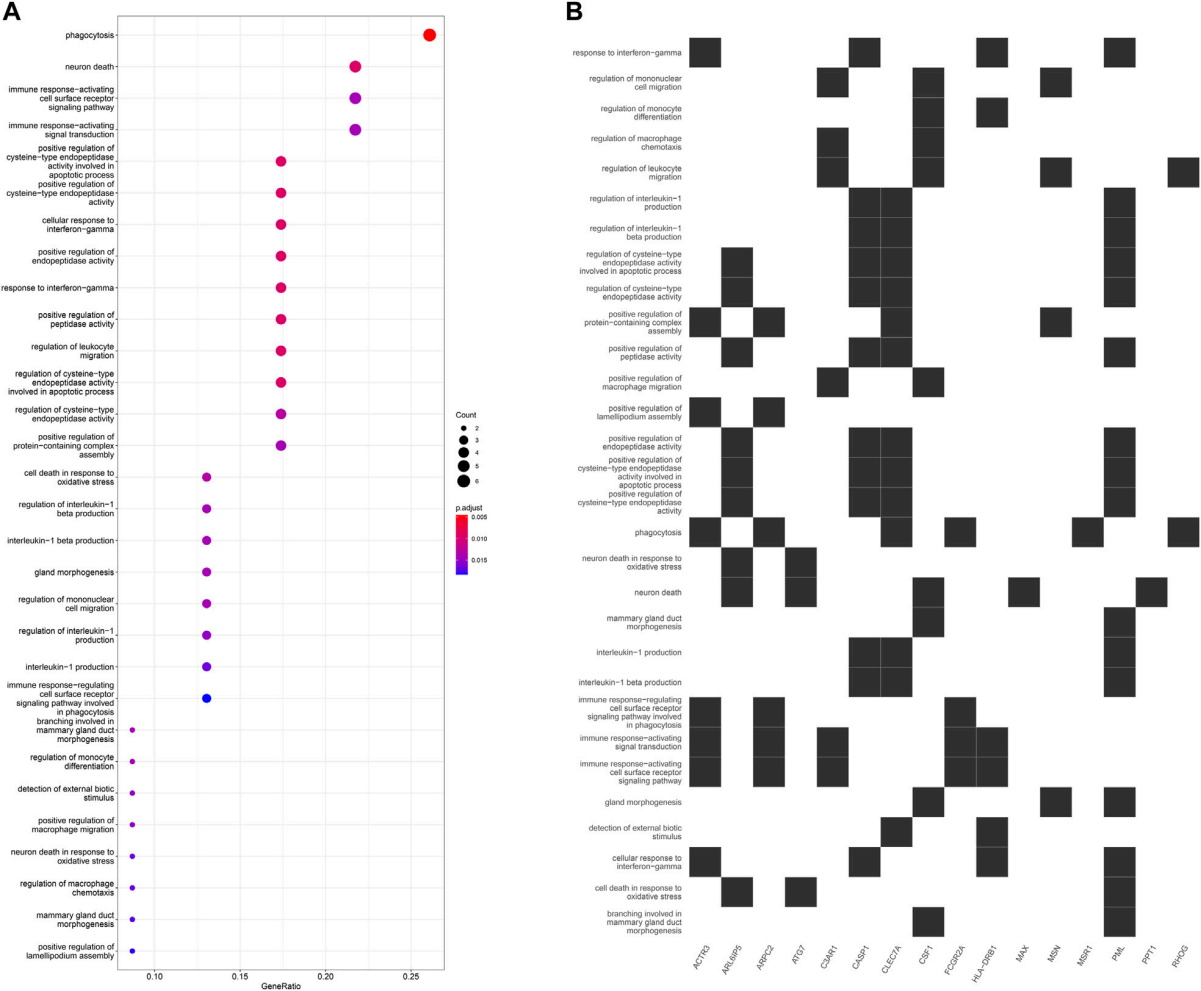
**(A)** Dot plot of enriched terms. Dot color reflects the *p* value; dot size, gene count; and position on x-axis, gene ratio. **(B)** Heatplot showing the complex associations between genes and gene sets.

GO enrichment analysis of the 24 hub genes confirmed that they had an immune-related function. A cluster tree plot of the top 30 GO bioprocesses showed the main pathway clusters: cellular detection biotic interferon−gamma, neuron death oxidative stress, immune assembly phagocytosis bioprocesses, gland migration morphogenesis duct, and interleukin−1 cysteine−type production endopeptidase (Figures 10A, 11A). Associations were observed between the genes and bioprocesses (Figures 10B, 11B), and the bioprocesses formed a network whose edges connected overlapping gene sets (Figure 10C). Most hub genes identified through single-cell RNA sequencing of HCC tissues were associated with immune cells (Supplementary Figure S9).

### Identification of Prognosis-Related Hub Genes and Development of a Prognostic Model

**FIGURE 12:**
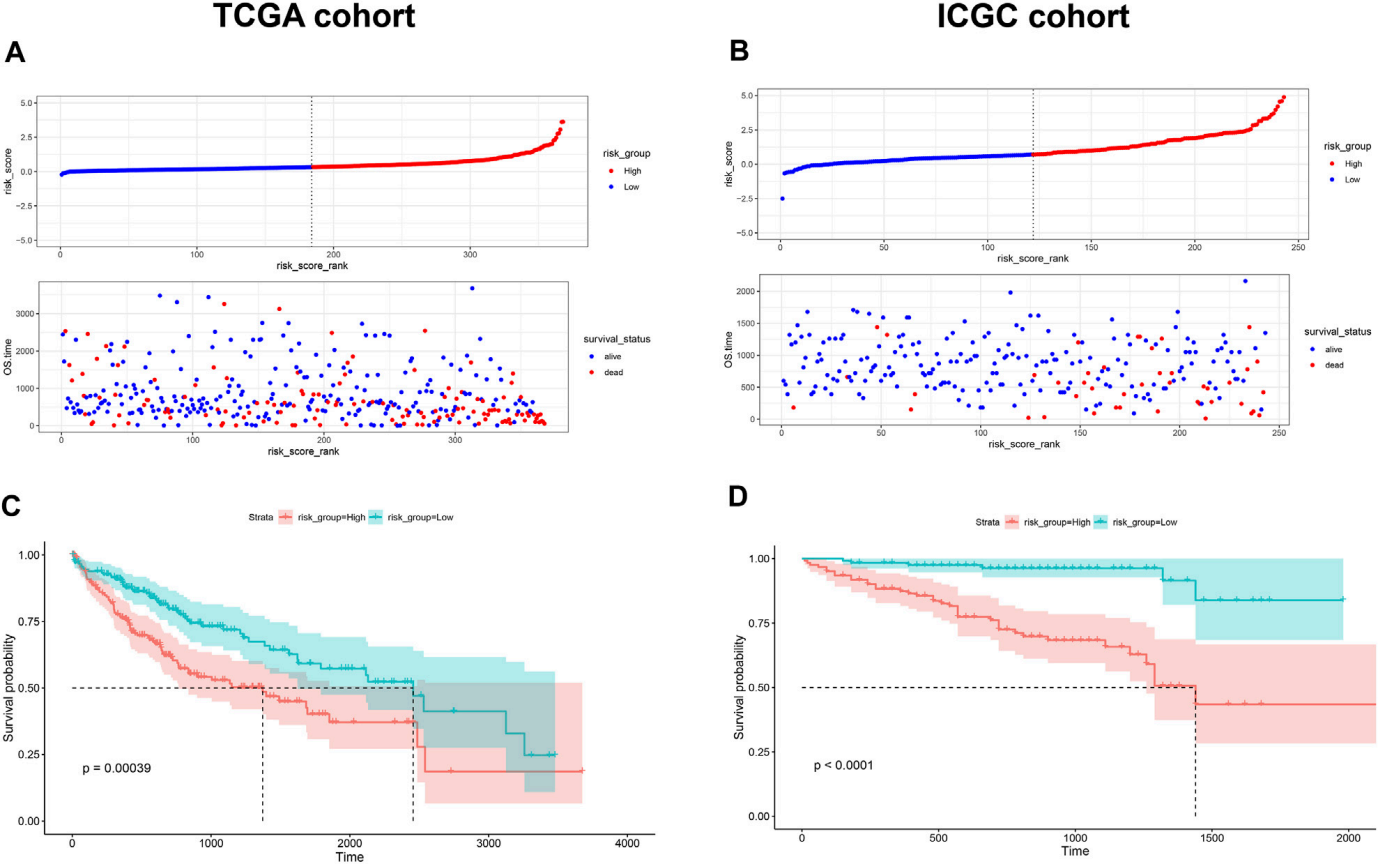
**(A,B)** Distribution of risk scores in the TCGA-LIHC and ICGC-LIRI-JP cohorts. Dotted vertical lines show the median risk score, which served as the cutoff for stratifying patients as low risk (blue dots) or high risk (red dots). Patients were also stratified as dead (red dots) or alive (blue dots). **(C,D)** Kaplan-Meier analysis of overall survival in the TCGA-LIHC and ICGC-LIRI-JP cohorts.

**FIGURE 13:**
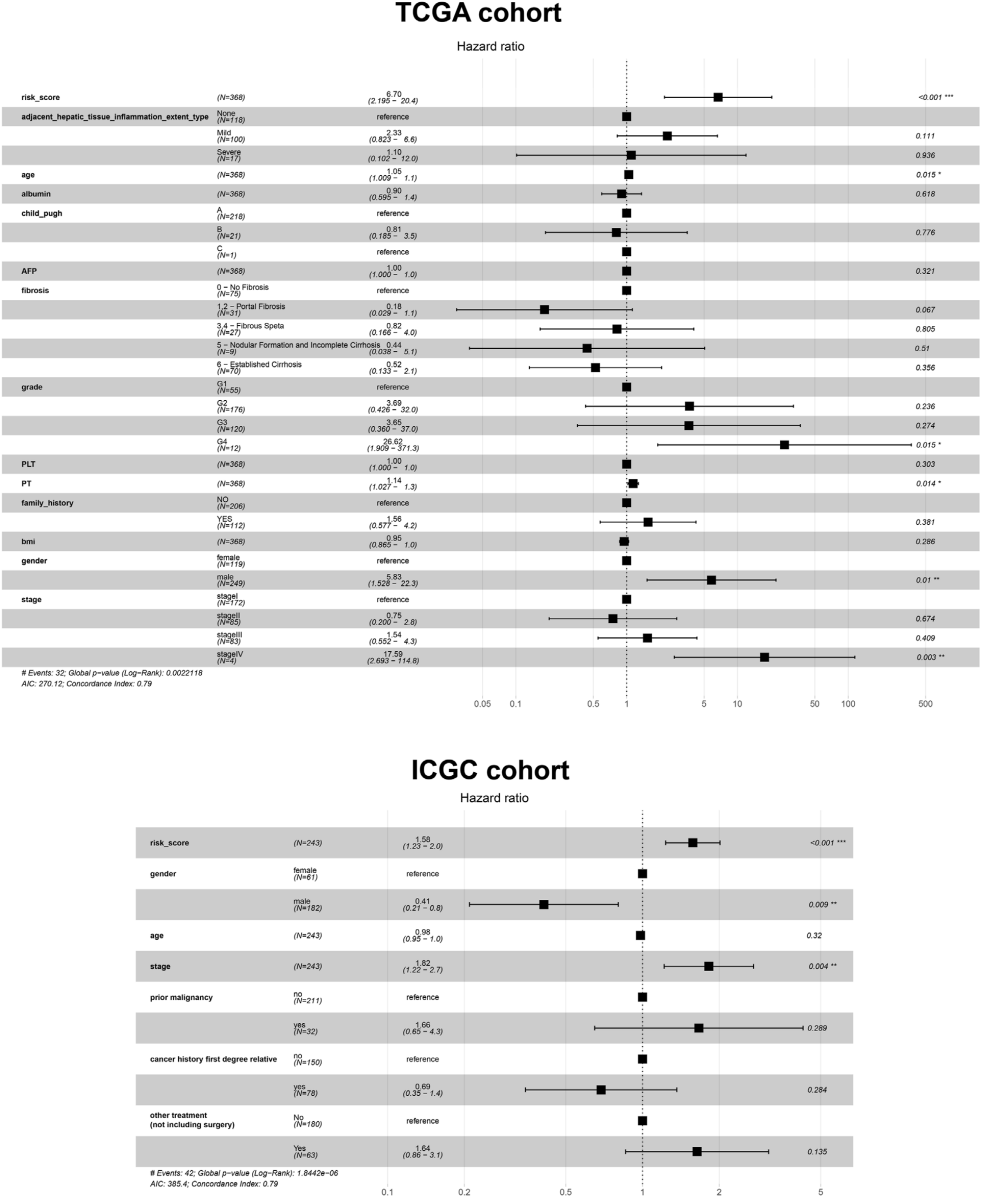
Cox regression analysis based on the risk score and other prognostic variables using data from the TCGA-LIHC and ICGC-LIRI-JP cohorts.

Given the prognostic potential of MEcyan, we performed univariable Cox regression on each hub gene. All hub genes met the assumption of proportional hazards (Supplementary Figure S10A). We tested *HJURP* and all hub genes in multivariable Cox regression models. Variables were selected using likelihood ratio forward selection. We built a significant Cox regression model using four genes: *HJURP*, *PPT1*, *PML*, and *CLEC7A.* With this model, we defined a risk score (Supplementary Figure S10B). Figures 12A,B show the distribution of risk scores and patient survival in the TCGA and ICGC cohorts. Low-risk groups showed significantly longer overall survival than high-risk groups (*p* < 0.01; Figures 12C,D). The prognostic risk score also proved to be an independent prognostic factor (*p* < 0.01; Figure 13).

**FIGURE 14:**
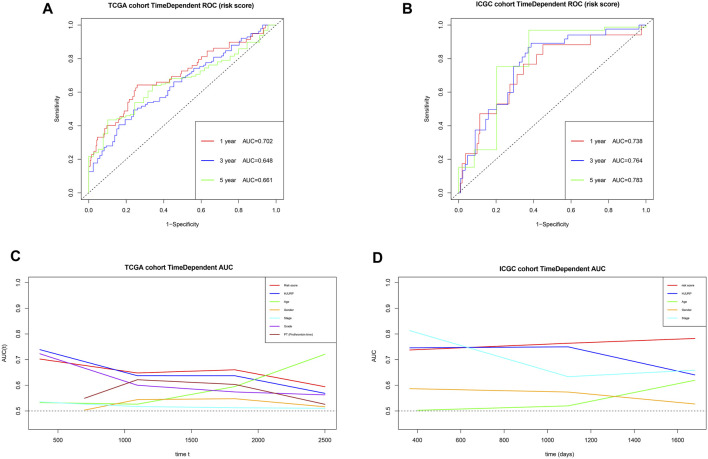
**(A,B)** Time-dependent receiver operating characteristic curves to assess the ability of the risk score to predict survival in the TCGA and ICGC cohorts. **(C,D)** Areas under the time-dependent receiver operating characteristic curves for the risk score, *HJURP* alone or other prognostic parameters, applied to the TCGA and ICGC cohorts.

Using the risk score and TCGA data, we obtained AUCs of 0.702 for 1-year survival, 0.648 for 3-year survival, and 0.661 for 5-year survival (Figure 14A). Next we validated the risk score externally using ICGC, obtaining AUCs of 0.738 for 1-year survival, 0.764 for 3-year survival and 0.783 for 5-year survival (Figure 14B). These results suggest that the risk score predicts survival of HCC patients moderately well. The risk score gave comparably good AUCs as other prognostic parameters in many cases, and it outperformed the other parameters for predicting long-term survival. In addition, the AUCs for the risk score appeared to be more stable than those for other prognostic parameters (Figures 14C,D).

We use the TCGA dataset as the training set to construct a random survival forest model. The random survival forest model constructed by *HJURP, PPT1, PML*, and *CLEC7A* has an out of bag (OOB) error of 0.41. Supplementary Figure S11A shows the relationship between OOB errors and the number of trees. We calculated the predicted values of the model for the training set (TCGA), plotted the TimeDependentROC curve and calculated the AUC, in which the 1-year survival AUC was 0.836; the 3-year survival AUC was 0.842; and the 5-year survival AUC was 0.892 (Supplementary Figure S11B). We calculated the predicted values of the model for the test set (LIHC). TimeDependentROC curves were used to assess the predictive performance of the model in the test set. The 1-year survival AUC in the test set was 0.726; the 3-year survival AUC was 0.748; and the 5-year survival AUC was 0.731 (Supplementary Figure S11C). The random survival forest model performed well in the training set, with AUC values above 0.8, outperforming the performance of the risk score based on Cox regression. However, its performance on the test set is significantly poorer than that on the training set, and its performance is slightly poorer than that of the risk score on the test set. Because only four features were used to construct the model, the robustness of the model was decreased, leading to overfitting of the model. Therefore, in addition to the risk score constructed based on Cox regression, random survival forest can also be used as another method to construct prognostic predictors. The other parameters of the random survival forest modfel are shown in Supplementary Table S5.

**FIGURE 15:**
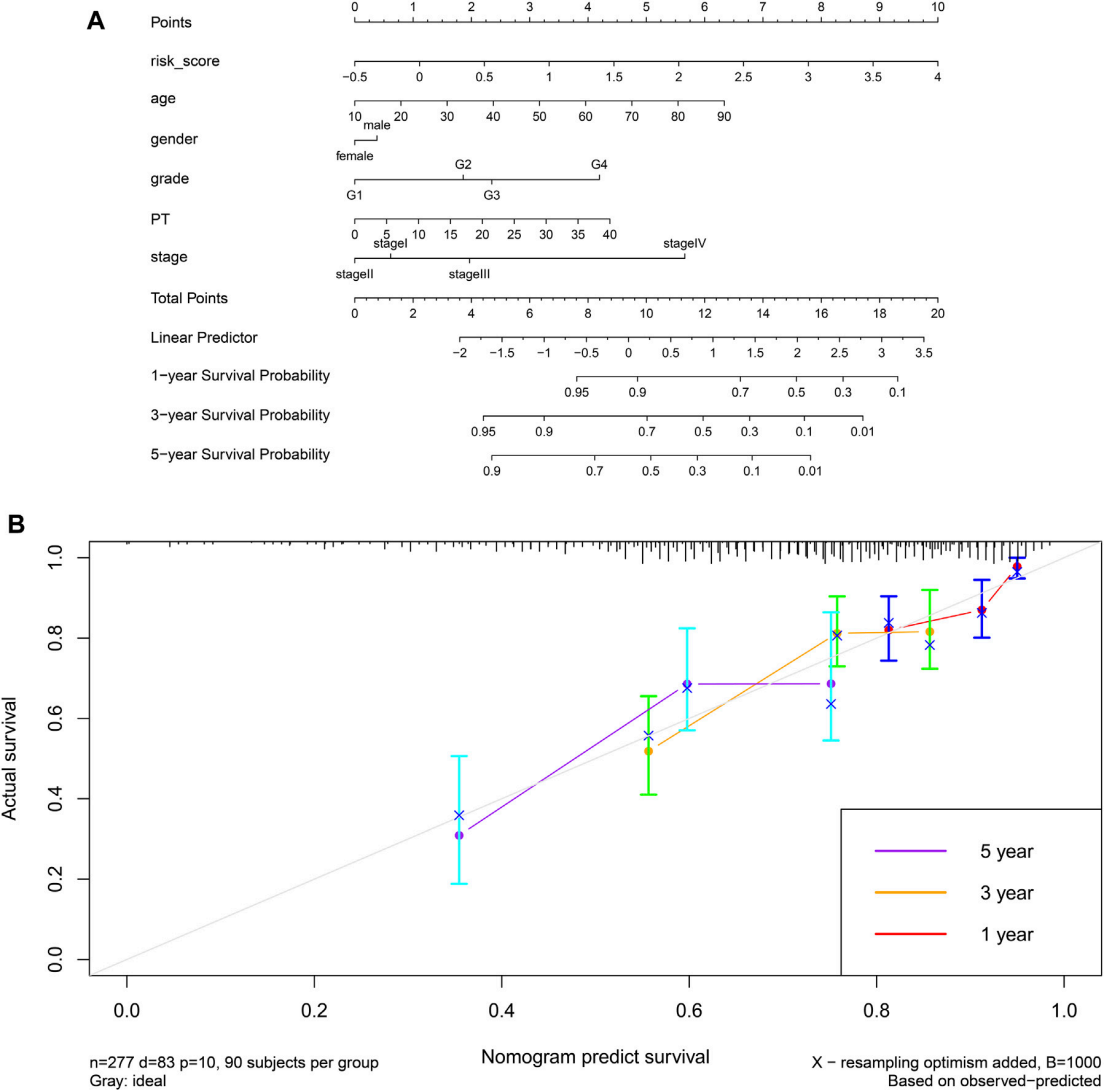
**(A)** A prognostic nomogram for HCC based on the TCGA-LIHC cohort. **(B)** Calibration curve for the nomogram.

Finally, we established a prognostic model based on risk score, age, sex, grade, prothrombin time and stage, and we constructed the corresponding nomogram (Figure 15A). Testing of the model against the TCGA cohort gave a C-index of 0.6631 and moderate fit for the calibration curve (Figure 15B). The prognostic model performed better than other prognostic parameters at predicting survival, particularly long-term survival (Supplementary Figure S11).

## Discussion

Our previous study showed that *HJURP* is overexpressed in HCC tissues, and that this overexpression is associated with worse survival (17). In the present work, we used GEPIA2 and TCGA-LIHC data to provide further evidence that *HJURP* is a proto-oncogene in HCC and an independent prognostic factor. We also show that *HJURP* may contribute to HCC by association with immune responses within the TME.

IOBR indicated that higher *HJURP* expression was associated with higher signature score for the cell cycle. This is consistent with the fact that *HJURP* is required for binding of CENP-A to centromeres during telophase/early G1 phase, which ensures proper chromosome separation during mitosis (8, 28). This role may help explain how *HJURP* promotes the proliferation of HCC cells (17, 29), through a mechanism involving activation of MAPK/ERK1/2 and AKT/GSK3β signaling pathways (13). We have further shown that polymorphism in *HJURP* influences risk of HCC among Chinese (30), and that the protein promotes HCC cell migration and invasion (17). These considerations strongly suggest that *HJURP* acts via the cell cycle to promote hepatocyte proliferation and act as a proto-oncogene.

IOBR further suggested a link between *HJURP* and other tumor signatures such as DDR, mismatch repair, and homologous recombination. To our knowledge, our study is the first to report such a link, which should be explored in future work.

Our IOBR analysis also linked *HJURP* to the TME in HCC. High *HJURP* expression was associated with higher risk of MDSC infiltration, promotion of immune checkpoints, and exhaustion of CD8^+^ T cells. Multiple algorithms linked *HJURP* expression to infiltration by B cells, various subtypes of T cells, and dendritic cells. *HJURP* has already been linked to the TME in other cancers. High tumor mutation burden in *HJURP* and other genes in prostate cancer is associated with greater activation of memory CD4^+^ T cells (31). *HJURP* has also been associated with the TME in clear cell renal cancer (32).

We explored correlations between *HJURP* expression and infiltration by specific types of immune cells initially based on bulk RNA sequencing, but we obtained greater resolution when we drew on single-cell sequencing data from normal liver and PBMCs. We found that *HJURP* was highly expressed in T cells, B cells and erythrocytes in normal liver; as well as in plasmablasts, Treg cells, exhausted memory B cells, memory CD4^+^ Th1 T cells and effector memory CD8^+^ T cells in PBMCs. *HJURP* was also highly expressed in CD8^+^ T cells, dendritic cells and a proportion of malignant hepatocytes in HCC tissues. Different subtypes of immune cells were associated with HJURP expression depending on whether the analysis was performed using bulk or single-cell sequencing data. Nevertheless, both types of analysis suggest that *HJURP* is expressed by B cells, T cells, and dendritic cells.

Our analyses link *HJURP* expression to immunosuppressive signatures. To understand whether the expression of *HJURP* in T cells be related to immune checkpoints, we compared *HJURP* expression in CD8^+^ T cell subtypes expressing different levels of PD-1. PD-1 is expressed in activated T cells, where it binds to a ligand and then inhibits T cell activation. PD-1 acts together with other signaling molecules in the TME to cause T cell depletion. We found that CD8^+^ T cells strongly expressing PD-1 also expressed high levels of *HJURP*. This finding suggests co-expression of *HJURP* and PD-1, and it implies that high *HJURP* expression in T cells coexpresses with immune checkpoints.

Consistent with this idea, *HJURP* was found to be expressed in dendritic cells and B cells. Dendritic cells are antigen-presenting cells that activate T cells but that can express the ligand for PD-1 and thereby suppress anti-tumor immune responses (33). B cells may also participate in immune checkpoints, although this needs to be explored in future research. Single-cell GSEA in our study linked *HJURP* overexpression in hepatocytes to downregulation of immune-related processes, which points to the deficiencies of immunogenicity of *HJURP* high expressing hepatocytes. Various reasons such as reduced immunogenicity and changes in energy metabolism of tumor cells can also lead to T cell dysfunction in the tumor microenvironment (34). This possibility also needs to be explored in future.

Indeed, our study identifies several aspects of *HJURP* that deserve further study, including the downstream effector molecules that mediate the observed associations between *HJURP* and HCC pathogenesis. *HJURP* has already been shown to regulate pathways involving GSK3β/JNK, p53, Wnt/β-catenin, MDM2/p53, YAP1/NDRG1, MAPK/ERK1/2, AKT/GSK3β, and SPHK1 (11–14, 35–37). Among these pathways, those involving MAPK/ERK1/2 and AKT/GSK3β have been associated with HCC proliferation (13), while SPHK1 signaling has been associated with the epithelial-to-mesenchymal transition in HCC (37). SPHK1 induces T cell failure and upregulates the PD-1 ligand, creating an immunosuppressive TME (38, 39). Similarly, activation of Wnt/β-catenin, MDM2/p53, and YAP1 signaling leads to immunosuppression, and blocking such signaling can improve the efficacy of ICIs (40–42).

We were able to demonstrate that the observed relationships between expression of *HJURP* and expression of immune-related genes may have immediate clinical potential as a way to predict survival of HCC patients. We identified three immune-related genes (*PPT1*, *PML*, *CLEC7A*) that, when combined with *HJURP,* allowed the definition of a risk score and random survival forest model. All three of these genes have previously been linked to cancer. *PPT1* can promote tumor growth and has already shown prognostic potential on its own in various cancer and HCC (43, 44). Downregulating *PPT1* can improve the efficacy of ICIs (45). *PML*, whose encoded protein is also known as TRIM19, can regulate various cytokine-induced signaling pathways (46). *CLEC7A* encodes dectin, which helps to form the TME and which can suppress CD4^+^ and CD8^+^ T cells in pancreatic cancer (47). High *CLEC7A* expression has been linked to poor survival in breast cancer (48). These previous studies suggest that our risk score and the underlying analysis are reliable.

Indeed, we were able to combine the risk score with other prognostic parameters to make a nomogram that showed moderate ability to predict patient survival. The nomogram itself may turn out to be a useful tool in the clinic, if its accuracy can be validated in future studies. In any event, our analysis strongly suggests that *HJURP* influences HCC patient survival through its coexpression with immune-related genes.

Our results should be interpreted with caution in light of the fact that our analyses were based entirely on bioinformatics and publicly available data. Future studies should verify experimentally whether *HJURP* can directly influence the response of HCC to immunotherapy. *In vitro* and animal studies are needed in order to elucidate themolecular mechanisms through which *HJURP* may contribute to an immunosuppressive TME.

Despite these limitations, our study provides strong evidence that *HJURP* is associated with the TME in HCC, and that genes related to *HJURP* and immune responses may affect the survival of HCC patients.

## Data Availability

Publicly available datasets were analyzed in this study. This data can be found here: TCGA-LIHC dataset: https://xenabrowser.net/datapages/?cohort=GDC%20TCGA%20Liver%20Cancer%20(LIHC)&removeHub=https%3A%2F%2Fxena.treehouse.gi.ucsc.edu%3A443ICGC-LIRI-JP; dataset: https://dcc.icgc.org/projects/LIRI-JP; GEO GSE111389 dataset: https://www.ncbi.nlm.nih.gov/gds/?term=GSE111389[Accession]; GEO GSE156625 dataset: https://www.ncbi.nlm.nih.gov/gds/?term=GSE156625%5BAccession%5D.
