# Supplementary figures and images for: Holliday Cross-Recognition Protein HJURP: Association With the Tumor Microenvironment in Hepatocellular Carcinoma and With Patient Prognosis
Source: Pathol Oncol Res. 2022 Jun 17;28:1610506. doi: 10.3389/pore.2022.1610506 (PMC9248293; doi:10.3389/pore.2022.1610506)

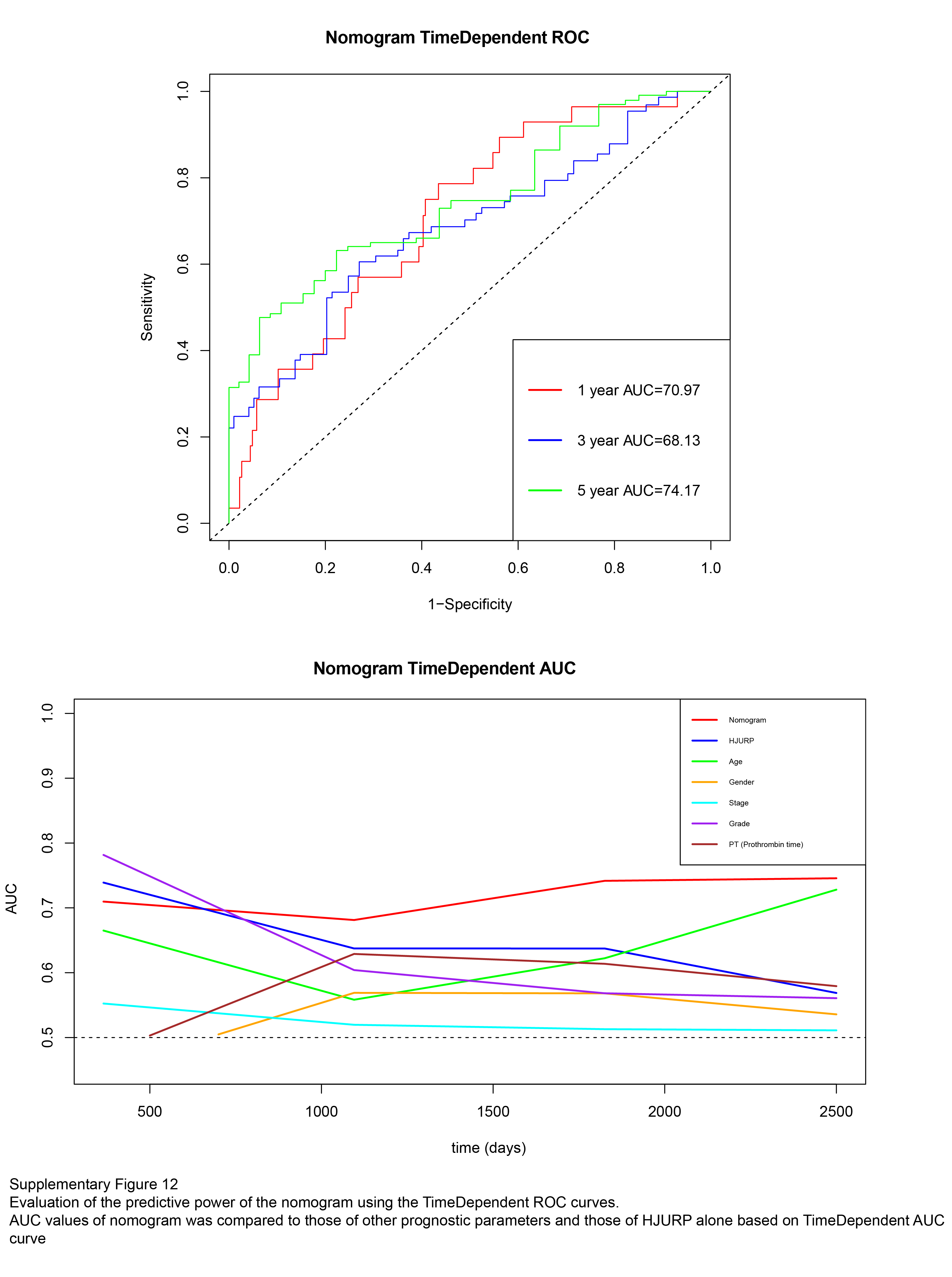

Supplement: Supplementary file 9 [file Image12.JPEG]

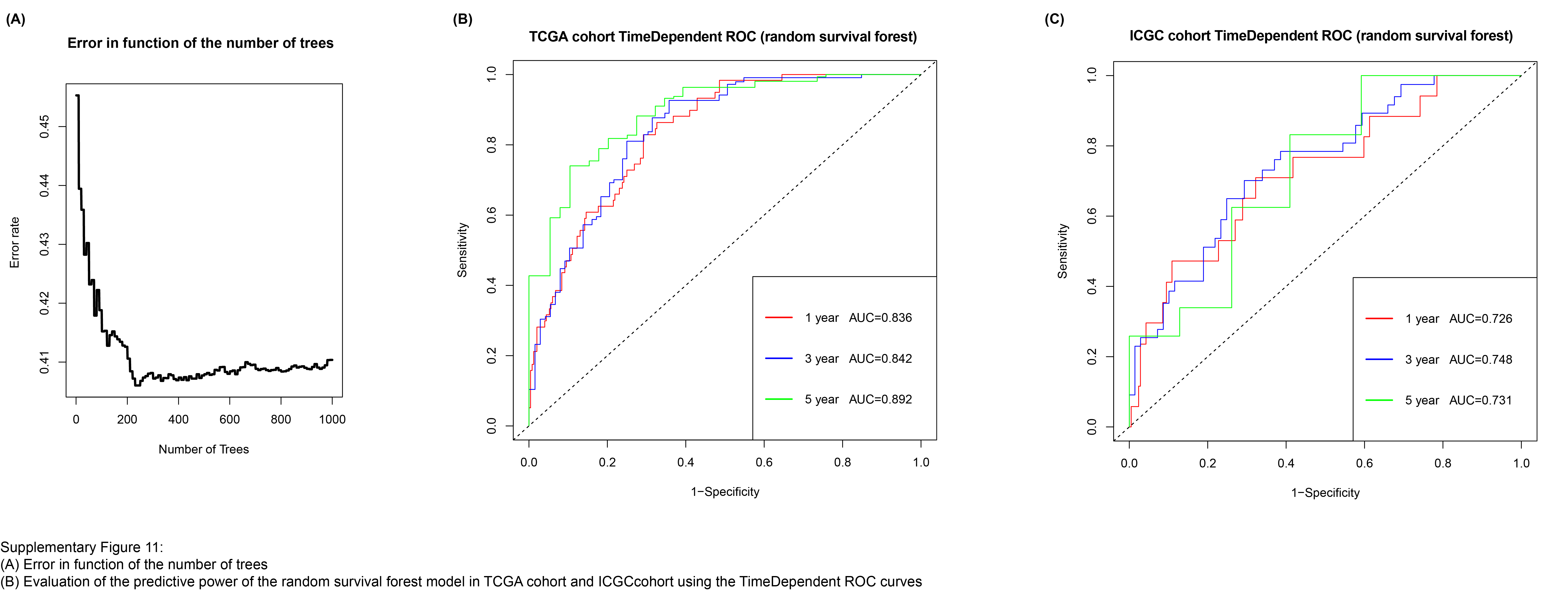

Supplement: Supplementary file 10 [file Image11.JPEG]
