# Supplementary material for: Holliday Cross-Recognition Protein HJURP: Association With the Tumor Microenvironment in Hepatocellular Carcinoma and With Patient Prognosis
Source: Pathol Oncol Res. 2022 Jun 17;28:1610506. doi: 10.3389/pore.2022.1610506 (PMC9248293; doi:10.3389/pore.2022.1610506)
Supplement: Supplementary file 12 [file DataSheet1.docx]

Supplementary table1. Part of clinical characteristics of tumor sample donors from TCGA-LIHC cohort

| characteristics | level | Overall |
| --- | --- | --- |
|  |  | 368 |
| adjacent hepatic tissue inflammation extent type (%) | Mild | 100 (42.6) |
|  | None | 118 (50.2) |
|  | Severe | 17 (7.2) |
| age (median [IQR]) | | 61.00 [52.00, 69.00] |
| albumin (median [IQR]) | | 4.00 [3.50, 4.30] |
| Child-pugh (%) | A | 218 (90.8) |
|  | B | 21 ( 8.8) |
|  | C | 1 ( 0.4) |
| AFP (median [IQR]) | | 14.50 [4.00, 260.50] |
| fibrosis (%) | 0 - No Fibrosis | 75 (35.4) |
|  | 1,2 - Portal Fibrosis | 31 (14.6) |
|  | 3,4 - Fibrous Speta | 27 (12.7) |
|  | 5 - Nodular Formation and Incomplete Cirrhosis | 9 ( 4.2) |
|  | 6 - Established Cirrhosis | 70 (33.0) |
| grade (%) | G1 | 55 (15.2) |
|  | G2 | 176 (48.5) |
|  | G3 | 120 (33.1) |
|  | G4 | 12 ( 3.3) |
| PLT (median [IQR]) | | 211.00 [160.25, 295.75] |
| post-operation ablation embolization therapy (%) | NO | 272 (91.6) |
|  | YES | 25 ( 8.4) |
| post-operation chemotherapy radiotherapy (%) | NO | 284 (94.7) |
|  | YES | 16 ( 5.3) |
| PT (median [IQR]) | | 1.10 [1.00, 9.15] |
| Family history (%) | NO | 206 (64.8) |
|  | YES | 112 (35.2) |
| bmi (median [IQR]) | | 24.57 [21.79, 28.67] |
| gender (%) | female | 119 (32.3) |
|  | male | 249 (67.7) |
| stage (%) | not reported | 24 ( 6.5) |
|  | stage I | 172 (46.7) |
|  | stage II | 85 (23.1) |
|  | stage III | 3 ( 0.8) |
|  | stage IIIa | 63 (17.1) |
|  | stage IIIb | 8 ( 2.2) |
|  | stage IIIc | 9 ( 2.4) |
|  | stage IV | 1 ( 0.3) |
|  | stage IVa | 1 ( 0.3) |
|  | stage IVb | 2 ( 0.5) |

*Abbreviation: Prothrombin Time (PT), platelet (PLT), Interquartile Range (IQR)*

Supplementary table2. Part of clinical characteristics of tumor sample donors from ICGC-LIRI-JP cohort

| characteristics | level | Overall |
| --- | --- | --- |
|  |  | 243 |
| gender (%) | female | 61 (25.1) |
|  | male | 182 (74.9) |
| age (median [IQR]) | | 69.00 [62.00, 74.00] |
| stage (%) | I | 36 (14.8) |
|  | II | 110 (45.3) |
|  | III | 76 (31.3) |
|  | IV | 21 ( 8.6) |
| prior malignancy (%) | no | 211 (86.8) |
|  | yes | 32 (13.2) |
| cancer history first-degree relative (%) | no | 150 (61.7) |
|  | unknown | 15 ( 6.2) |
|  | yes | 78 (32.1) |
| treatment type (%) | chemotherapy | 2 ( 0.8) |
|  | no treatment | 180 (74.1) |
|  | other therapy | 58 (23.9) |
|  | surgery | 3 ( 1.2) |

*Abbreviation: Interquartile Range (IQR)*

Supplementary table3. Cell clusters annotated by marker genes, which was identified by Wilcoxon test

| Cell type | cluster | Marker gene | P val | avg_log2FC | pct.1 | pct.2 | P val adj |
| --- | --- | --- | --- | --- | --- | --- | --- |
| T cells | T cells | CD3E | 0 | 1.146373 | 0.446 | 0.172 | 0 |
|  |  | CD3D | 0 | 1.410199 | 0.662 | 0.231 | 0 |
|  | CD4+ T | LTB | 0 | 1.056316 | 0.365 | 0.161 | 0 |
|  |  | CCR7 | 4.69E-99 | 0.537643 | 0.125 | 0.053 | 1.19E-94 |
|  |  | IL7R | 0 | 1.494507 | 0.63 | 0.199 | 0 |
|  | CD8+ T | CD8B | 5.23E-89 | 0.369121 | 0.298 | 0.093 | 1.32E-84 |
|  |  | CXCL13 | 2.35E-161 | 0.952244 | 0.128 | 0.015 | 5.96E-157 |
|  | Treg | IL7R | 1.99E-108 | 0.304295 | 0.45 | 0.22 | 5.03E-104 |
|  |  | FOXP3 | 0 | 1.259505 | 0.227 | 0.007 | 0 |
|  |  | TIGIT | 0 | 2.157674 | 0.627 | 0.081 | 0 |
| B cells | B | MZB1 | 0 | 4.077907 | 0.937 | 0.018 | 0 |
|  | naive B | BANK1 | 0 | 1.848333 | 0.323 | 0.003 | 0 |
|  |  | MS4A1 | 0 | 2.911853 | 0.519 | 0.007 | 0 |
| NK cells | NK1 | FGFBP2 | 0 | 2.687191 | 0.424 | 0.029 | 0 |
|  |  | MYOM2 | 0 | 1.544779 | 0.147 | 0.009 | 0 |
|  |  | GZMB | 0 | 3.31524 | 0.715 | 0.088 | 0 |
|  |  | GZMH | 0 | 2.423604 | 0.578 | 0.107 | 0 |
|  | NK2 | KLRF1 | 1.41E-141 | 0.511989 | 0.165 | 0.062 | 3.56E-137 |
|  |  | CD7 | 0 | 1.109171 | 0.585 | 0.215 | 0 |
|  |  | NKG7 | 0 | 1.872934 | 0.845 | 0.295 | 0 |
|  |  | CMC1 | 8.81E-105 | 0.470499 | 0.344 | 0.204 | 2.23E-100 |
|  |  | CXCL2 | 6.00E-59 | -1.18504 | 0.018 | 0.089 | 1.52E-54 |
| Monocyte-derived cells | TAM | C1QB | 0 | 4.467378 | 0.504 | 0.025 | 0 |
|  |  | C1QA | 0 | 4.410806 | 0.518 | 0.024 | 0 |
|  |  | CD163 | 0 | 1.092553 | 0.197 | 0.002 | 0 |
|  | DC | CLEC9A | 2.59E-219 | 0.734779 | 0.176 | 0.005 | 6.55E-215 |
|  |  | CD1C | 1.02E-274 | 1.563842 | 0.262 | 0.009 | 2.59E-270 |
|  |  | CLEC10A | 2.46E-135 | 0.881954 | 0.251 | 0.016 | 6.23E-131 |
| Endothelial cells | Endothelial cells | VWF | 0 | 2.873595 | 0.336 | 0.037 | 0 |
|  |  | PECAM1 | 0 | 2.42967 | 0.346 | 0.081 | 0 |
|  |  | PLPP1 | 0 | 1.939265 | 0.265 | 0.079 | 0 |
|  | CD9+ endo | CD9 | 0 | 2.294242 | 0.648 | 0.116 | 0 |
|  |  | CAV1 | 0 | 2.735093 | 0.606 | 0.08 | 0 |
|  | PLVAP+ endo | PLVAP | 0 | 2.928346 | 0.884 | 0.055 | 0 |
| Malignant hepatocytes | Hepatocyte4 | APOA2 | 6.70E-10 | -1.29056 | 0.429 | 0.327 | 1.69E-05 |
|  |  | APOC3 | 0 | 0.592587 | 0.63 | 0.262 | 0 |
|  | Hepatocyte1 | SPINK1 | 0 | 2.852241 | 0.618 | 0.067 | 0 |
|  | Hepatocyte2 | FABP1 | 4.45E-116 | 0.645094 | 0.392 | 0.143 | 1.13E-111 |
|  | Hepatocyte3 | C3 | 3.86E-64 | 1.034074 | 0.141 | 0.076 | 9.78E-60 |
|  |  | FGA | 2.78E-116 | 0.954129 | 0.179 | 0.083 | 7.03E-112 |
|  |  | FGB | 5.59E-128 | 0.593139 | 0.222 | 0.106 | 1.42E-123 |
| Fibroblast cells | Fibroblast | ACTA2 | 0 | 5.886914 | 0.864 | 0.022 | 0 |
|  |  | MYL9 | 0 | 5.16922 | 0.915 | 0.029 | 0 |
|  |  | TAGLN | 0 | 5.930294 | 0.913 | 0.022 | 0 |
| Bi-potent cells | Bi-potent | KRT19 | 0 | 4.674478 | 0.667 | 0.007 | 0 |
|  |  | TNFRSF12A | 0 | 3.632838 | 0.664 | 0.044 | 0 |

*Abbreviation: Regulatory T Cells (Treg), Natural Killer Cells (NK cells), Tumor-Associated Macrophage (TAM), Dendritic Cell (DC), Endothelial cells (endo), P value (P val), average log2(fold change) (avg log2FC), percent (pct), P value adjustment (P val adj)*

Supplementary table4. Cell clusters highly expressing HJURP

| *gene* | *cluster* | *P val* | *avg log2FC* | *pct.1* | *pct.2* | *P val adj* |
| --- | --- | --- | --- | --- | --- | --- |
| HJURP | Hepatocytes | 0.00 | 0.42 | 0.09 | 0.00 | 0.00 |
| HJURP | CD8+ T cells | 0.00 | 0.49 | 0.13 | 0.00 | 0.00 |
| HJURP | Dendritic cells | 0.00 | 0.27 | 0.17 | 0.01 | 0.00 |

*Abbreviation: P value (P val), average log2(fold change) (avg log2FC), percent (pct), P value adjustment (P val adj)*

Supplementary table5. The parameters of the random survival forest model

| Sample size: | 368 |
| --- | --- |
| Number of deaths: | 131 |
| Number of trees: | 1000 |
| Forest terminal node size: | 15 |
| Average number of terminal nodes: | 17.95 |
| Number of variables tried at each split: | 2 |
| Total number of variables: | 4 |
| Resampling used to grow trees: | sampling without replacement |
| Resample size used to grow trees: | 233 |
| Analysis: | RSF |
| Family: | survival |
| Splitting rule: | logrank *random* |
| Number of random split points: | 10 |
| (OOB) CRPS: | 0.20199909 |
| (OOB) Requested performance error: | 0.41037053 |

*Abbreviation: random survival forest (RSF), out of bag (OOB), continuous rank probability score (CRPS)*

­­
